# Are allergic diseases and internalizing and externalizing behaviours in children related? A cross-sectional study

**DOI:** 10.1192/j.eurpsy.2022.696

**Published:** 2022-09-01

**Authors:** E. Gonzalez-Fraile, M.P. Berzosa-Grande, R. Sánchez-López, M. Soria-Oliver, S. Rueda-Esteban

**Affiliations:** 1 Universidad Internacional de La Rioja, Faculty Of Health Sciences, Madrid, Spain; 2 International University of La Rioja, Department Of Health Sciences, Madrid, Spain; 3 Intelecto Psychological Centre, Therapy, Jeréz de la Frontera, Spain; 4 Universidad Pública de Navarra, Health Sciences, Pamplona, Spain; 5 San Carlos Clinic Hospital, Respiratory, Madrid, Spain

**Keywords:** Child, behaviour, parent, allergy

## Abstract

**Introduction:**

The prevalence of allergies in children has grown in last few decades. Allergies are very often associated with physical, mental, and emotional problems that could be detected through child’s behaviour and feelings.

**Objectives:**

to describe and compare children’s behaviour (internalizing and externalizing) across a sample of children aged 6–11 years with and without allergic diseases.

**Methods:**

This was a cross-sectional observational case-control study. A survey to 366 families (194 allergic cases and 172 controls), including a child behaviour checklist (CBCL) and a socio-demographic questionnaire with questions related to family, school education, health conditions and allergy symptoms, was administered.

**Results:**

Children with a diagnosis of allergy showed higher scores in the overall CBCL score (standardised mean differences [SMD]= 0.47; confidence intervals [CI]: 0.26–0.68) and in the internalizing and externalizing factors (SMD=0.52 and SMD=0.36, respectively) than non-allergic children. Odds ratio (OR) analyses showed a higher risk (OR=2.76; 95% CI [1.61 to 4.72]) of developing a behavioural difficulty in children diagnosed with allergies. Age and level of asthma appear as modulatory variables.

**Conclusions:**

Children aged 6–11 years diagnosed with allergies showed larger behavioural problems than non-allergic children. This relationship is stronger in internalizing behaviours. These findings suggest the importance of attending to them and treating them in the early stages of diagnosis to avoid future psychological disorders.

**Disclosure:**

No significant relationships.

